# Persistent Effectivity of Gas Plasma-Treated, Long Time-Stored Liquid on Epithelial Cell Adhesion Capacity and Membrane Morphology

**DOI:** 10.1371/journal.pone.0104559

**Published:** 2014-08-29

**Authors:** Maxi Hoentsch, René Bussiahn, Henrike Rebl, Claudia Bergemann, Martin Eggert, Marcus Frank, Thomas von Woedtke, Barbara Nebe

**Affiliations:** 1 Department of Cell Biology, University Medical Center Rostock, Rostock, Germany; 2 Leibniz-Institute for Plasma Science and Technology e.V., Greifswald, Germany; 3 Center for Extracorporeal Organ Support, Department of Internal Medicine, University Medical Center Rostock, Rostock, Germany; 4 Medical Biology and Electron Microscopic Center, University Medical Center Rostock, Rostock, Germany; University Paul Sabatier, France

## Abstract

Research in plasma medicine includes a major interest in understanding gas plasma-cell interactions. The immediate application of gas plasma in vitro inhibits cell attachment, vitality and cell-cell contacts via the liquid. Interestingly, in our novel experiments described here we found that the liquid-mediated plasma effect is long-lasting after storage up to seven days; i. e. the liquid preserves the characteristics once induced by the argon plasma. Therefore, the complete Dulbecco's Modified Eagle cell culture medium was argon plasma-treated (atmospheric pressure, kINPen09) for 60 s, stored for several days (1, 4 and 7 d) at 37°C and added to a confluent mouse hepatocyte epithelial cell (mHepR1) monolayer. Impaired tight junction architecture as well as shortened microvilli on the cell membrane could be observed, which was accompanied by the loss of cell adhesion capacity. Online-monitoring of vital cells revealed a reduced cell respiration. Our first time-dependent analysis of plasma-treated medium revealed that temperature, hydrogen peroxide production, pH and oxygen content can be excluded as initiators of cell physiological and morphological changes. The here observed persisting biological effects in plasma-treated liquids could open new medical applications in dentistry and orthopaedics.

## Introduction

Plasma medicine has emerged as one of the most recent developments to be actually based on plasma physics and chemistry. Plasma medicine is a new area of interdisciplinary research combining biology, chemistry and physics [1], [2]. The numbers of fields finding application in medicine are increasing and include e. g. blood coagulation [1] and coblation surgery [3], bio-decontamination [4]–[6], wound healing [7], stimulation of tissue regeneration [8], [9] but interestingly, also contrary outcome, e. g. antiproliferative and antitumorigenic effects [10]. Effects of atmospheric gas plasmas on cancer cell signaling were recently discovered, e. g. PI3K/AKT inhibition by the elevated levels of cellular reactive oxygen species (ROS) [11]. There is rapidly growing worldwide interest especially in the development and application of atmospheric pressure low-temperature plasma devices for biomedical applications [12]. The application of gas plasma may have different effects on living matter. The lethal effect of plasma on various bacteria species has been studied extensively in recent years [13], [14]. Investigations of Fröhling *et al*. [15] showed that even a short course of time is sufficient to inactivate the gram-positive *Listeria innocua* and the gram-negative *Escherichia coli*. An extensive investigation of Ermolaeva *et al*. [16] also showed bactericidal effects of non-thermal plasma. A significant reduction of various pathogenic bacteria was shown in, inter alia, *Pseudomonas aeruginosa*, *Escherichia coli*, *Staphylocoocus aureus*, *Enterococcus faecium* and *Burkholderia cenocepacia*. In this connection, the study of Ermolaeva *et al*. [16] as well showed that gram-negative bacteria were more susceptible to non-thermal plasma treatment than gram-positive bacteria and bacteria colonies in biofilms were less sensitive to non-thermal plasma treatment. Non-thermal gas plasmas as a therapeutic treatment option against various cancer cell lines have yielded promising results. Low-temperature plasma killed leukemia cells with increasing doses of exposure using a plasma pencil [17]. Recent studies reported antiproliferative effects of a low-temperature plasma jet (helium flow in open air) in HCT116 colon carcinoma cells growing in a 3D environment using multi-cellular tumor spheroids [10].

Furthermore, cell physiological processes can be induced by plasma treatment that lead to increased tissue healing [18]–[20] but without disturbing e. g. the skin barrier or moisture [21]. In the healing process of tissues, the adhesion molecules of the cells play a crucial role for the cells' adhesion, migration and proliferation and are therefore, responsible for many cell functions [22]–[24]. Integrins as transmembrane heterodimeric adhesion receptors are connected via their extracellular domain to extracellular matrix proteins in the surrounding tissue [25], [26]. In this connection, Haertel *et al*.. [27] recently revealed that plasma treatment leads to changes in the plasma membrane for phosphatidylserine residues (Annexin-V test) as well as in integrin receptor expression in HaCaT keratinocytes, which was accompanied by an increased level of intracellular reactive oxygen species. Reactive nitrogen and reactive oxygen species (RNS, ROS) are attributed a possible key role for the plasma effects on living matter [12].

One of the key findings of basic research in plasma medicine of the last recent years is that several biological effects are mainly mediated by the liquid environment surrounding the cells [28]. Simple liquids like water or physiological saline, respectively, develop antimicrobial activity after treatment by atmospheric pressure plasma. These effects are attributed to the generation of different low-molecular reactive species [29]–[33]. Furthermore, plasma treatment of more complex liquids like cell culture media could induce various effects on living cells and their components [34]–[38]. Plewa *et al.* have shown quite recently that plasma preconditioned media experienced the same antiproliferative effects on tumor cells as cells immersed in culture media exposed to the low-temperature plasma [10].

Regarding our previous work, the application of atmospheric pressure argon plasma inhibits attachment and vitality of epithelial cells suspended in a complete cell culture medium, whereby these effects amplify with increasing treatment time up to 120 s [34]. Furthermore, we observed the solely medium-mediated effect on cellular adhesion structures called tight junctions in mHepR1 epithelial cells after plasma treatment. The ZO-1 protein, which exhibits a characteristic marker for tight cell-cell contacts, was adversely affected and large openings could be recognised.

Based on this, in the present work we focused on determining whether argon plasma-treated, complete cell culture medium stored for one week is as effective for cell physiology as when applied immediately. For these experiments an atmospheric pressure argon plasma jet was used and murine liver epithelial cells mHepR1 were observed for the tight junction protein ZO-1, cell surface morphology, long-time cell adhesion and respiration. Furthermore, cell culture medium was analysed for changes in molecular oxygen content, pH value and hydrogen peroxide. The working hypothesis behind this research is if plasma-treated liquids additionally stored for longer time-periods are sufficient to induce biological effects.

## Experimental design

### 1. Argon plasma source

The experiments were carried out using the atmospheric pressure plasma jet (APPJ) kINPen 09. The APPJ source consists of a quartz capillary (inner diameter of 1.6 mm) in the middle of which a needle electrode (diameter of 1 mm) is mounted. A high-frequency (HF) voltage of 1.1 MHz/2–6 kV is applied at this electrode. Argon gas (purity >99.996%) is used as feed gas with a gas flow of 1.9 slm. Despite dry gas is supplied out of the bottle, gas tubings may gather air humidity due to permeation and condensation effects in phases when the plasma jet is not operated and the tubings are not flushed. Gas humidity may influence significantly the effect of plasma treatment on cells [39]. In order to reduce the variation of gas humidity, the plasma jet was allowed to warm-up for at least 15 min before experiments were started. Typically, the feed gas humidity after this period is less than 100 ppm. A gas discharge ignites at the tip of the high voltage needle, excites the argon gas and leads to the generation of a low temperature plasma which is blown out of the capillary. The so-called plasma jet outside the nozzle has a length of 12–14 mm and is about 1 mm wide. The temperature at the visible tip of the plasma jet does not exceed 50°C. The gas temperature has been measured by means of a fibre optic thermometer (Luxtron FOT Lab Kit + STF probe), placed end-on in the effluent of the plasma jet. The plasma radiation has been spectroscopically analysed by using a compact spectrometer (Avantes AvaSpec-3648) which was equipped with a cosine corrector. The latter was protected by a quartz disk from the direct influence of the plasma. In order to determine the UV-irradiance on a surface in a certain distance to the jet nozzle, the system was calibrated against a PTB traceable deuterium halogen lamp (Avantes AVAlight-DH-S-BAL). Particulars for the characterisation of the APPJ are given in Weltmann *et al.* [9].

### 2. Argon plasma treatment of cell culture medium

**Figure 1:**
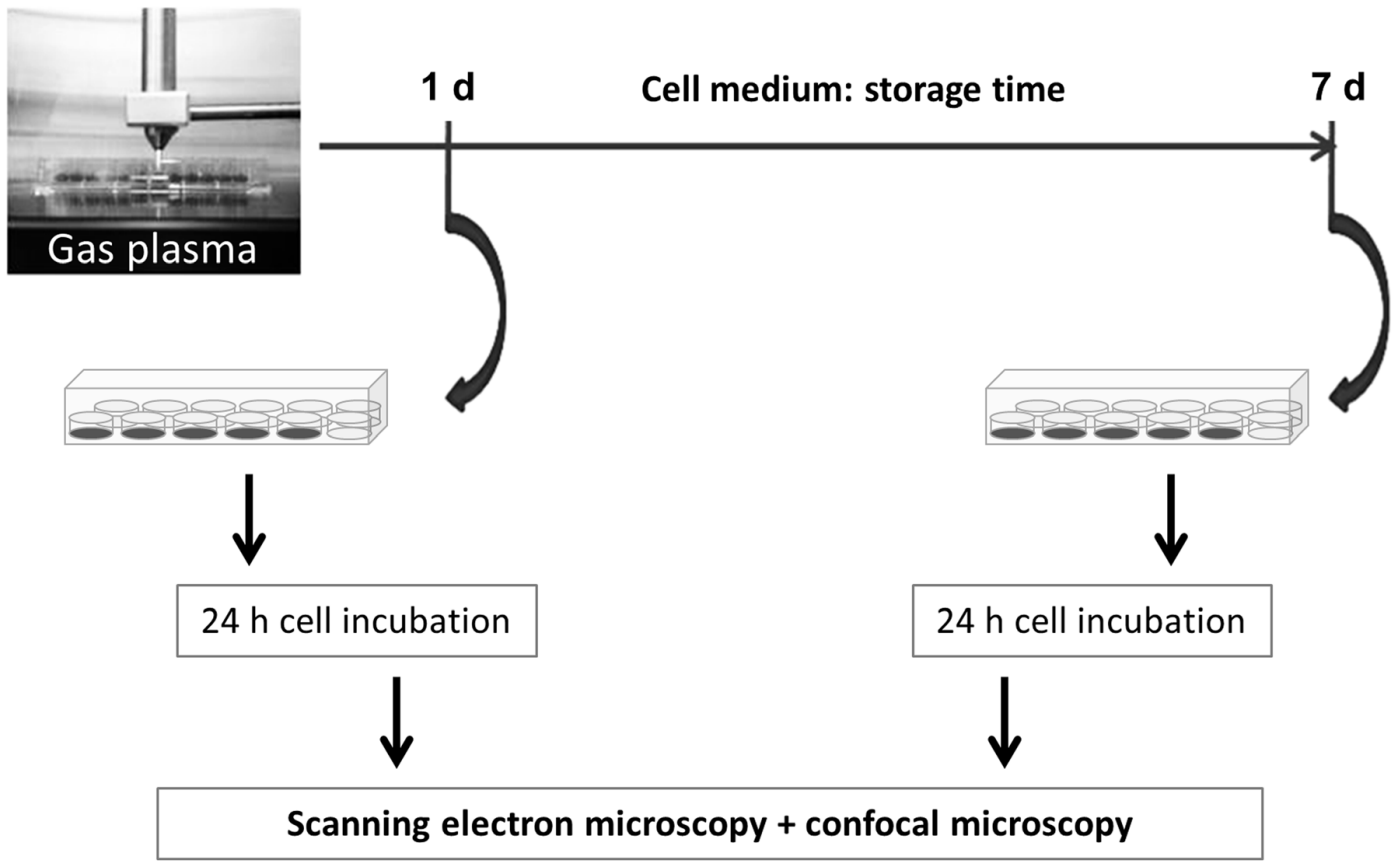
Workflow. Cell biological investigations after cultivation with argon plasma-treated, long-time stored cell medium DMEM.

All experiments were conducted following the same procedure (**Figure 1**). Taking into consideration that the plasma effect is mediated by liquids [10], [28], [34]–[38], only the cell culture medium was treated with argon plasma or for some experiments (pH, oxygen) only with argon gas. For the argon plasma treatment, the front end of the quartz capillary was positioned at the height of the top edge of a 96-well plate so the plasma had immediate contact with the cell culture medium. Each well of a 96-well plate was provided with 100 µl DMEM (Dulbecco's Modified Eagle Medium, liquid, high glucose, Invitrogen) supplemented with 10% FBS (Fetal Bovine Serum “Gold”, PAA Laboratories) and 1% gentamicin (Ratiopharm) and treated with argon plasma for 60 s in general, with the exception of ZO-1 experiments with additional 120 s. For the investigations of the long-lasting effect of plasma-treated DMEM on the tight junction protein ZO-1 and the cell membrane structure, the plasma-treated DMEM was first allowed to stand for 1 and 7 days (storage at 37°C and 5% CO_2_) before the plasma-treated DMEM was added to the mHepR1 monolayer (see 3.). Then, mHepR1 cells were incubated with the plasma-treated DMEM for 24 h at 37°C and 5% CO_2_ followed by preparations for immunofluorescence staining of ZO-1 (see 4.) and scanning electron microscopy (SEM, see 5.). For cell adhesion and respiration experiments, the plasma-treated DMEM was pooled (see procedure in Figure 1, final amount for the flow system: 50 ml) and first allowed to stand for 4 days at 37°C and 5% CO_2_ before adding to the mHepR1 monolayer on the metabolic chips (see 6.). To exclude negative thermal effects, the temperature was directly measured in DMEM during plasma treatment (see Figure 1, left image) using the fiber optic thermometer. The temperature probe was placed through a hole in the wall of a well at the bottom of it. The hole was sealed by means of adhesive tape. This optical sensor technology is based on the fluorescence decay time of a special thermo-sensitive phosphorescent (phosphor) sensor, to the end of a fiber optic probe.

### 3. Cell culture

The epithelial cell line mHepR1 (murine hepatocytes) [40] was used throughout the experiments. The immortalised mHepR1 cells represent a clone of the HepSV40 line derived from transgenic mice [40] and exhibits characteristic markers for epithelial cells like the tight-junction-associated protein ZO-1. The epithelial cells were cultured in DMEM (Dulbecco's Modified Eagle Medium, liquid, high glucose, Invitrogen) supplemented with 10% FBS (Fetal Bovine Serum “Gold”, PAA Laboratories) and 1% gentamicin (Ratiopharm) at 37°C and 5% CO_2_. The mHepR1-monolayer in a cell culture flask was detached enzymatically by 0.25% trypsin/0.38% ethylenediamine tetraacetic acid (Invitrogen). After trypsinisation (trypsinisation process was stopped by the addition of complete medium) the cell number was determined by a Neubauer counting chamber (Brand) and 5×10^4^ cells/well were seeded into a 24-well plate (Greiner), which was provided with a round cover slip (diameter of 12 mm, MENZEL) and filled up to a total volume of 1 ml per well using complete DMEM as described. The mHepR1 cells were incubated for 2 d at 37°C and 5% CO_2_ to achieve a confluency of nearly 100% for ZO-1 and microvilli observations.

### 4. Immunofluorescence staining of ZO-1

The long-lasting plasma effect on the zonula occludens (ZO-1) tight-junction protein was investigated.

For this purpose, 1 ml of fresh DMEM was treated with plasma in a 96-well plate (20×100 µl, Greiner) using the treatment times of 60 s and 120 s followed by a standing time of 1 and/or 7 days. The mHepR1 cells were seeded into a 24-well plate (5×10^4^cells/well) 2 days before expiry of the standing time of the plasma-treated DMEM. After achieving a confluency of nearly 100% within these 2 days, the old DMEM was removed and the plasma-treated DMEM or control medium was added to the mHepR1 monolayer. These cells were subsequently incubated for 24 h at 37°C and 5% CO_2_ followed by the immunofluorescence staining of the ZO-1 protein.

The cells were permeabilised with ice-cold acetone (−20°C, 200 µl, Lab-Scan) for 5 min and, after washing three times with phosphate buffer solution (PBS) (PAA Laboratories), the cells were incubated with rabbit anti-ZO-1 antibody (diluted 1∶100, 100 µl, Invitrogen) for 30 min at room temperature. Afterwards, the cells were again washed three times with PBS and incubated with fluorescein-conjugated Alexa Fluor 488 secondary antibody goat-anti-rabbit (1∶100, 100 µl, Invitrogen) for 30 min at room temperature in the dark to avoid a fading of the fluorescent dye. Finally, after washing (3×) with PBS, the cells were embedded on slides with fluorescence mounting medium (Eukitt). The ZO-1 protein was analysed by an inverted light microscope (AxioObserver.Z1, Carl Zeiss) and images were taken by AxioCamMRm.

### 5. Preparation for scanning electron microscopy

The plasma treatment preceding the investigations of the cell surface structure followed the same experimental procedure as for the ZO-1 studies (see Figure 1). After the 24 h incubation period with the plasma-treated DMEM at 37°C and 5% CO_2_, the mHepR1 monolayer was washed three times with PBS and subsequently fixed in 0.5% glutardialdehyde (GDA) (SIGMA-ALDRICH) in PBS. In order to accomplish the formation of highly cross-linked, insoluble protein aggregates the samples were stored for at least 24 h at 4°C. Afterwards the mHepR1 monolayer was critical point dried. For this purpose the GDA-PBS solution was first gradually removed by drainage using acetone with increasing concentrations of 30, 50, 70, 90 and 100% for 10 s, 5, 10, and 15 min and twice for 10 min, respectively. Subsequently, the acetone was substituted by critical point drying (K 850 EMITECH) and then samples were sputter coated with gold particles for 100 s (layer thickness ca 20 nm), achieving a conductive dissipation for scanning electron microscopy (SEM). The cell surface structure was analysed with a DSM 960 A (Carl Zeiss), operated at 10 kV and with a FE-SEM Merlin VP compact (Carl Zeiss), operated at 5 kV for high magnification imaging (15,000×) of microvilli. The images were imported into the iTEM imaging software (version 5.0, Olympus Soft Imaging Solutions) for measurements of microvilli length (in µm) using the calibrated iTEM free line measurement tool in a picture overlay mode. Nearly 100 microvilli per image (n = 5) were finally measured and the corresponding data were exported into Microsoft Excel worksheets for statistical analysis.

### 6. Continuous measurements of adhesion and respiration

The Bionas 1500 analysing system with the SC 1000 metabolic chips (Bionas GmbH) was described earlier [41]. This chip type is equipped with IDES (interdigitated electrode structures) sensors for cell adhesion and Clark-type sensors for respiration. For cell adhesion observations on living cells, the impedance was measured to detect insulating cell membranes near the measuring electrodes. Sinusoidally oscillating fields with a frequency of 10 kHz are used [42]. The insulating cell membrane of intact cells on the electrodes and their distance to the electrodes determine the current flow and thus the sensor signal. When the membranes are destroyed or cell-cell contacts are opened, there are less obstacles for an oscillating electrical field compared to the situation with intact cells. Thus higher capacitance and resistance values indicate the presence of damaged or opened cell membranes on the IDES.

The chips were decontaminated with ethanol and washed with PBS (PAA Laboratories) prior to the experiments. 150,000 cells were allowed to grow on the chips prior to the experiment for 24 h at 37°C and 5% CO_2_. The technology is based on a flow system, where fresh medium (in our experiments DMEM with 10% FBS, argon plasma-treated) is steadily supplied to the cells. To prevent contamination, the medium racks were sealed with a sterile membrane (BREATHseal, Greiner Bio-One) which could be penetrated easily by the aspiration needle. During the entire course of the experiment a pump phase of 4 min was followed by a steady phase (no flow) of 4 min. The flow rate in the system was adjusted to the lowest value of 56 µl/min to avoid shear stress. The temperature in the operating chamber was 37°C. After 24 h the flow medium was changed to 1% Triton X-100 (Sigma-Aldrich) diluted in PBS to disintegrate the cell membranes. The values obtained at this time point were referred to as 0% adhesion and respiration, respectively. 100% adhesion and respiration corresponds to the values of capacitance of intact cells just before the addition of the argon plasma-treated medium. Cell adhesion was calculated using the program Bionas analyser (Bionas 1500 Data Analyser, Version 1.07). For further processing of the values the software Microsoft Excel (Microsoft Office 2007) was used.

### 7. Measurements of hydrogen peroxide, pH and oxygen in the medium

To exclude the possibility of the long-lasting plasma effect of DMEM on cell physiology being caused by a change in the pH-value, hydrogen peroxide formation or a decrease of oxygen in the medium, these three parameters were evaluated.

*pH*: Fresh, complete DMEM (2 ml) was plasma-treated in a 96-well plate (20×100 µl) for 60 s, pooled and analysed by a pH-meter (SevenEasy METTLER TOLEDO) directly after plasma treatment as well as 1 d after plasma treatment. The pH measurements were conducted three times.

*H_2_O_2_*: The concentration of hydrogen peroxide was determined semi-quantitatively using peroxide test strips (Quantofix MACHERY-NAGEL). 100 µl of fresh, complete DMEM was plasma-treated for 60 s in a 96-well plate and the concentration of hydrogen peroxide was measured after 0, 0.5, 1, 2 and 24 h. The measurements of the concentrations of hydrogen peroxide in the 60 s plasma-treated DMEM were carried out 7 times.

*O_2_*: The oxygen content in the medium after plasma treatment was measured by an optical needle-type oxygen microsensor (Oxygen Micro-Optode, Type PSt1; Presens) connected to the Microx TX3 (Presens). For this purpose, 3×100 µl of fresh, complete DMEM was plasma-treated for 60 and 120 s in a 96-well plate. Medium was pooled from 3 wells and oxygen was quantified in the middle of the fluid volume. Experiments for plasma treatment and O_2_ measurement were repeated three times. The medium was stored for 1 and 24 h at 37°C and 5% CO_2_.

### 8. Gel filtration chromatography

Gel filtration chromatography is a method to separate molecules according to size. Gel-filtration experiments were performed on a GE-Healthcare high-performance liquid chromatography (HPLC) system (Freiburg, Germany) at a constant flow rate of 0.5 ml/min for 50 min. Separation was carried out on a Superdex 200 column (GE-Healthcare, Freiburg, Germany) using phosphate buffer (200 mM NaCl, 1.25 mM NaH_2_PO_4_, 3.5 mM Na_2_HPO_4_) as a separation buffer. The sample protein concentration was measured according to Bradford [43] and samples were diluted with distilled water to a concentration of 4 mg/ml. Fresh, complete DMEM (1 ml) was plasma-treated in a 96-well plate (20×100 µl) for 60 s and pooled. For gel filtration analysis, 500 µl of the sample was applied by injection. In the resulting histogram the large molecules appear in the first peak; the smaller the molecules, the more they are positioned to the right.

### 9. Statistics

Statistical analyses were performed with SPSS-software version 15.0 for Windows (SPSS Inc., Chicago). The unpaired *t*-test was used for samples with normal distribution (pretest: Kolomogorov-Smirnov), otherwise the Mann-Whitney *U*-test (H_2_O_2_ analysis). Data were presented as a mean ± a standard error of the mean (SEM). Results of p<0.05 were considered significant (*p<0.05; **p<0.01; ***p<0.001).

## Results

**Figure 2:**
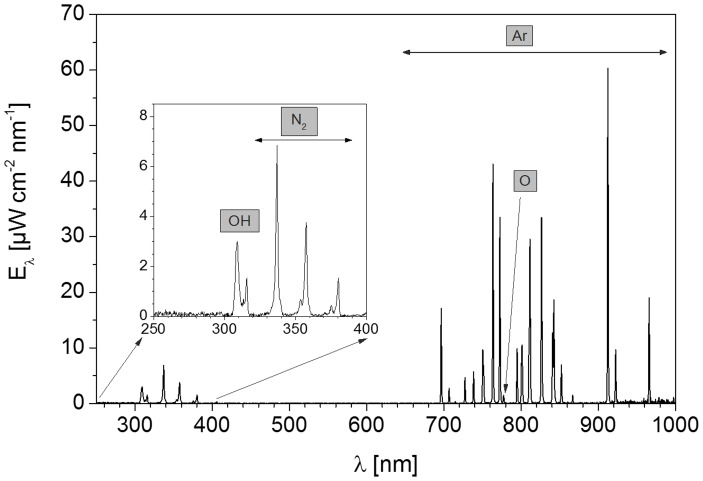
Overview spectrum of the argon plasma source at a gas flow rate of 1.9 slm. Notice the emission peak of reactive species of hydroxyl radicals at a wavelength of 310 nm. The emission lines at a wavelength of 690 nm are generated by the inert gas argon.

The spectral analysis of the used argon plasma source is shown in **Figure 2**. Possible biological hazards due to the UV radiation (300–380 nm) could be excluded, since UV-A and UV-B radiation are generated at a low intensity. In recent studies using keratinocytes it could be demonstrated, that UV radiation delivered by APPJ treatment is not the main route of plasma-cell interaction [44]. Close to a wavelength of 310 nm, an emission peak of hydroxyl radicals (OH) is seen. The peaks in the wavelength range of 313–400 nm resulted from the emission of molecular nitrogen. In the visible (380–780 nm) and near-infrared region (>780 nm), emission lines of argon were revealed, starting at wavelengths higher than 690 nm.

**Figure 3:**
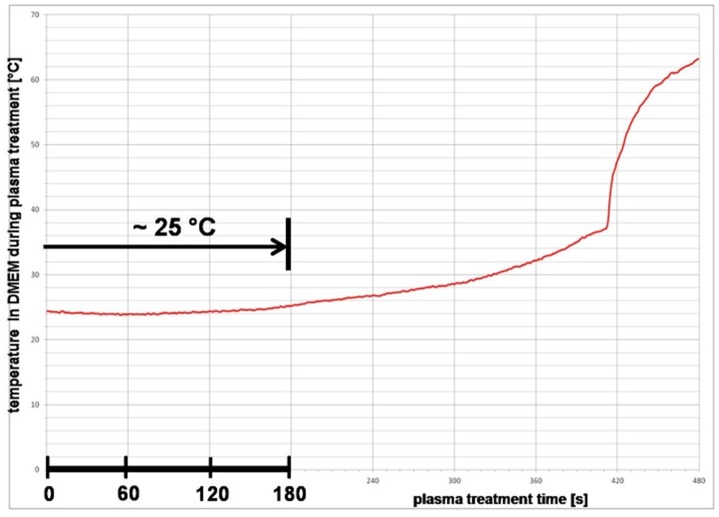
Argon plasma treatment and medium temperature. The average temperature in DMEM during plasma treatment up to 120 s is 25°C and remains constant up to a plasma treatment time of 180 s. Thus, negative thermal effects in the plasma-treated cell culture medium DMEM are excluded.

To exclude negative thermal effects in the medium, temperature measurements during plasma treatment were conducted. As shown in **Figure 3**, the temperature in the DMEM averages 25°C and remains constant up to a plasma treatment time of 180 s.

**Figure 4:**
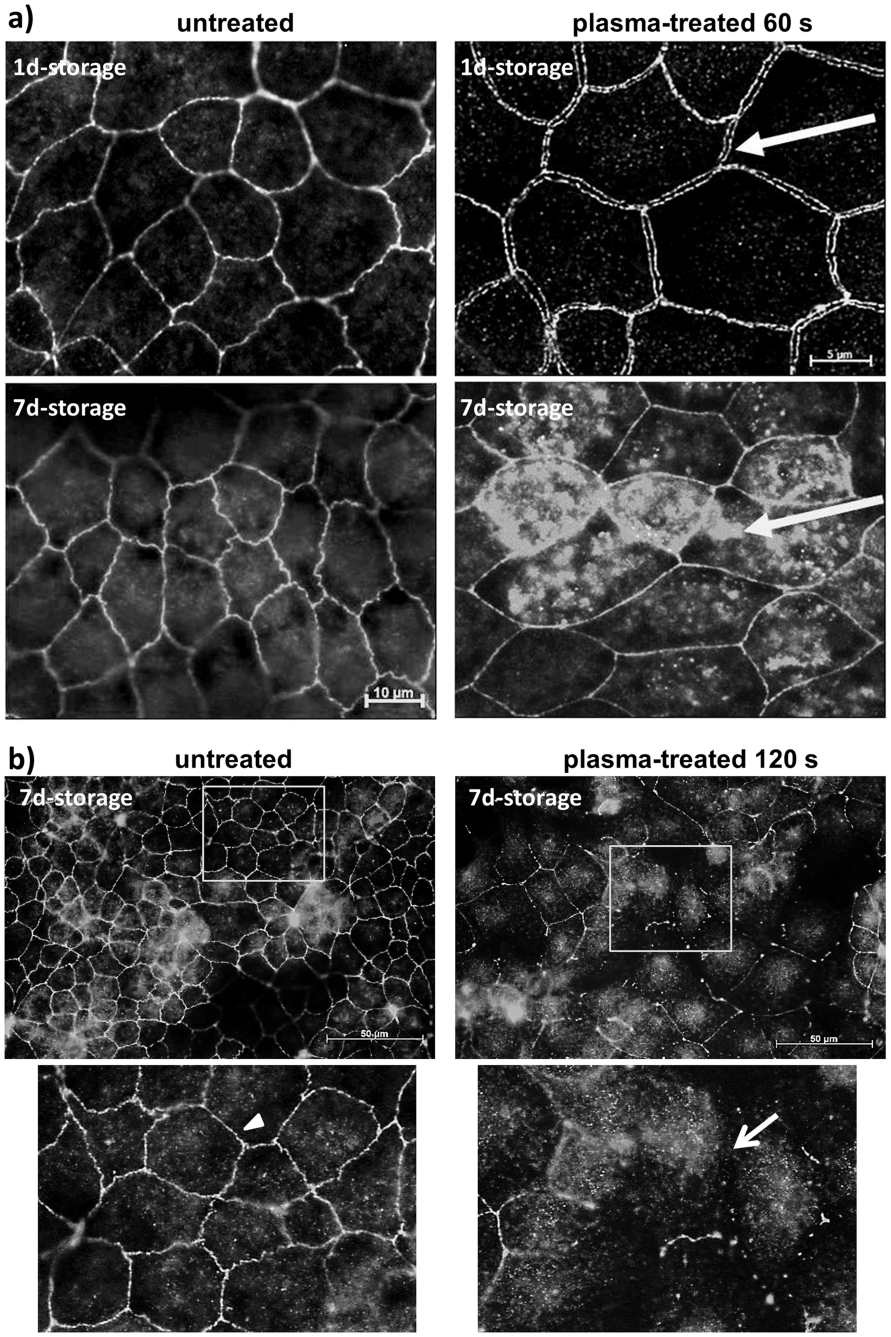
Tight junction architecture in the mHepR1 epithelial cell monolayer. *a)* Note the continuous ZO-1 bands between mHepR1 cells in normal, untreated cell medium DMEM (left column). In contrast, 60 s plasma-treated DMEM stored for 1 d induces large openings between cell margins (arrow, right above), indicating a break of tight junctions. Plasma-treated DMEM (60 s) stored for 7 d induces an irregular cytoplasmic distribution of the ZO-1 proteins (arrow, right below). (AxioObserver.Z1, Carl Zeiss, bars = 5, 10 µm). *b)* The plasma treatment time of 120 s of the DMEM and storage of 7 d induces not only a retraction of the ZO-1 protein into the cytoplasm but also a nearly complete loss of the cell-cell contacts (arrow), while normal cells show a strong bonding (arrowhead). Note the long-lasting argon plasma effect of cell medium on structural organisation of cells. (AxioObserver.Z1, bar = 50 µm, the windows indicate the areas of the magnified view below).

In our recent work [34], we found out that plasma effects on cells correspond to the plasma-medium interaction and thus plasma-treated DMEM alone is able to open cell-cell contacts in a confluent cell monolayer of mHepR1. As a result of these findings, the question arose of how long-lasting atmospheric pressure plasma-treated DMEM affects cell physiology and morphology, e. g. the tight junctions between the mHepR1 cells. For this purpose, the DMEM was plasma-treated for 60 and 120 s, stored for 1 and 7 days and then added to the mHepR1 monolayer followed by a subsequent cultivation period of 24 h and immunofluorescence staining of ZO-1 (see Figure 1). Normally, untreated cells present clear, continuous ZO-1 bands between adjoining cells throughout the whole cell monolayer as shown in **Figure 4**. In contrast, large intercellular openings were observed between the adjoining cells when plasma-treated DMEM (60 s) was stored up to 1 day before adding it to the mHepR1 monolayer (Figure 4a). Interestingly, even after 7 days of storage these plasma-mediated effects on tight junctions were observed, exhibiting an irregular distribution of the ZO-1 protein running centripetally (60 s) as well as a complete loss of the cell-cell contacts (120 s) (Figures 4a,b). The whole spectrum of tight junction alterations of one cell monolayer due to the 120 s plasma-treated medium is to be seen in Figure 4b ranging from a complete loss of cell-cell contacts (as indicated by the arrow) until a centripetal retraction of the ZO-1 protein in the cytoplasm or showing some fragmented tight junctions.

**Figure 5:**
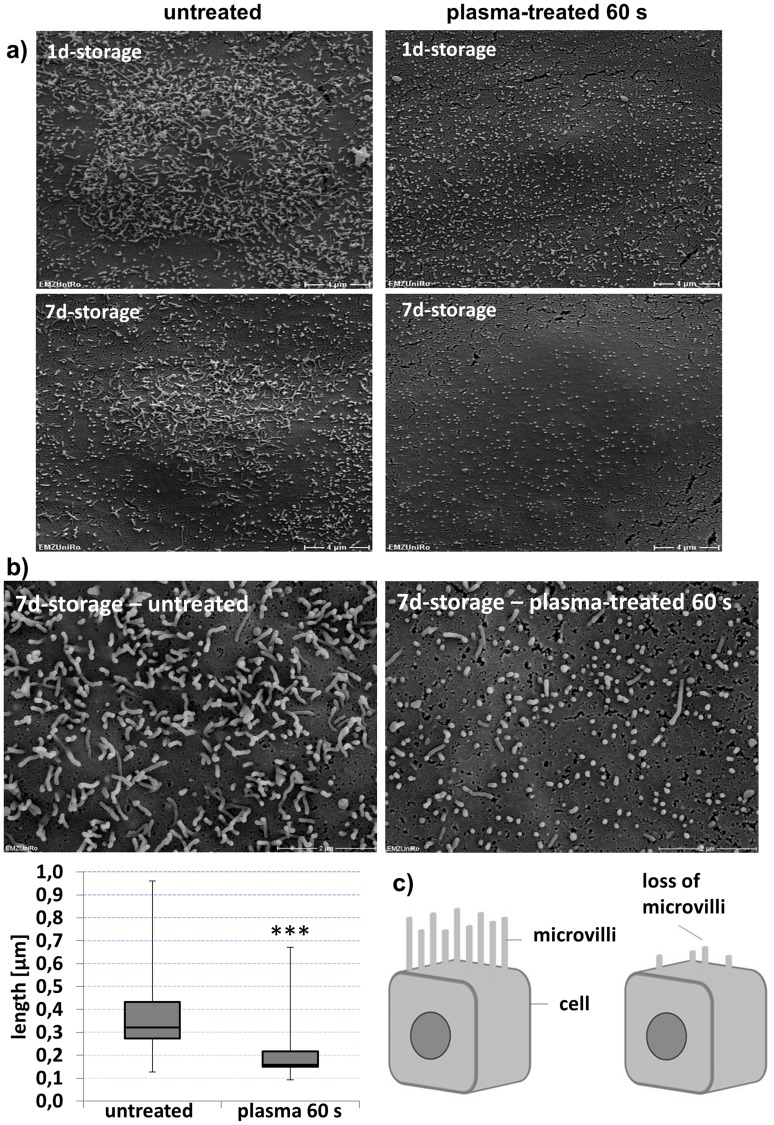
Cell membrane morphology of mHepR1 cells. *a)* The surface structure of cells after 24 h in normal, untreated medium (left column) presents elongated microvilli with high densities, which extend over the entire cell surface. In contrast, the mHepR1 cells in 60 s plasma-treated medium (right column) show extremely shortened microvilli accompanied by a reduced density (bar = 4 µm, 5,000× magnification, SEM DSM 960 A, Carl Zeiss). *b)* The significantly shortened microvilli are impressively visible using a higher magnification (bar = 2 µm, 15,000× magnification, FE-SEM Zeiss Merlin VP compact, Carl Zeiss) (p<0.001, student's *t-*test, n = 410–500 microvilli). *c)* Scheme: microvilli at the cell surface in normal epithelial cells (left) and cells exposed to plasma-treated DMEM (right).

Because cell morphology has an impact on cell physiology we investigated how the cell surface structure is affected by plasma-treated DMEM. To this end, the DMEM was plasma-treated for 60 s and was allowed to stand for 1 and 7 days. After completion of the standing time, the plasma-treated DMEM was added to the mHepR1 monolayer, incubated for 24 h and the cells were prepared for SEM. Untreated mHepR1 cells present elongated microvilli (median 0.32 µm in length) covering the cell surface with a high density (**Figure 5**), which was even visible on argon-treated cells (not shown). In contrast, the plasma-treated mHepR1 cells show significantly shortened microvilli (median 0.16 µm) (p<0.001, *t*-test, n = 410–500 microvilli) and their density over the entire cell surface is reduced. Interestingly, the plasma-treated DMEM shows a long-lasting effect also on the cell surface structure.

**Figure 6:**
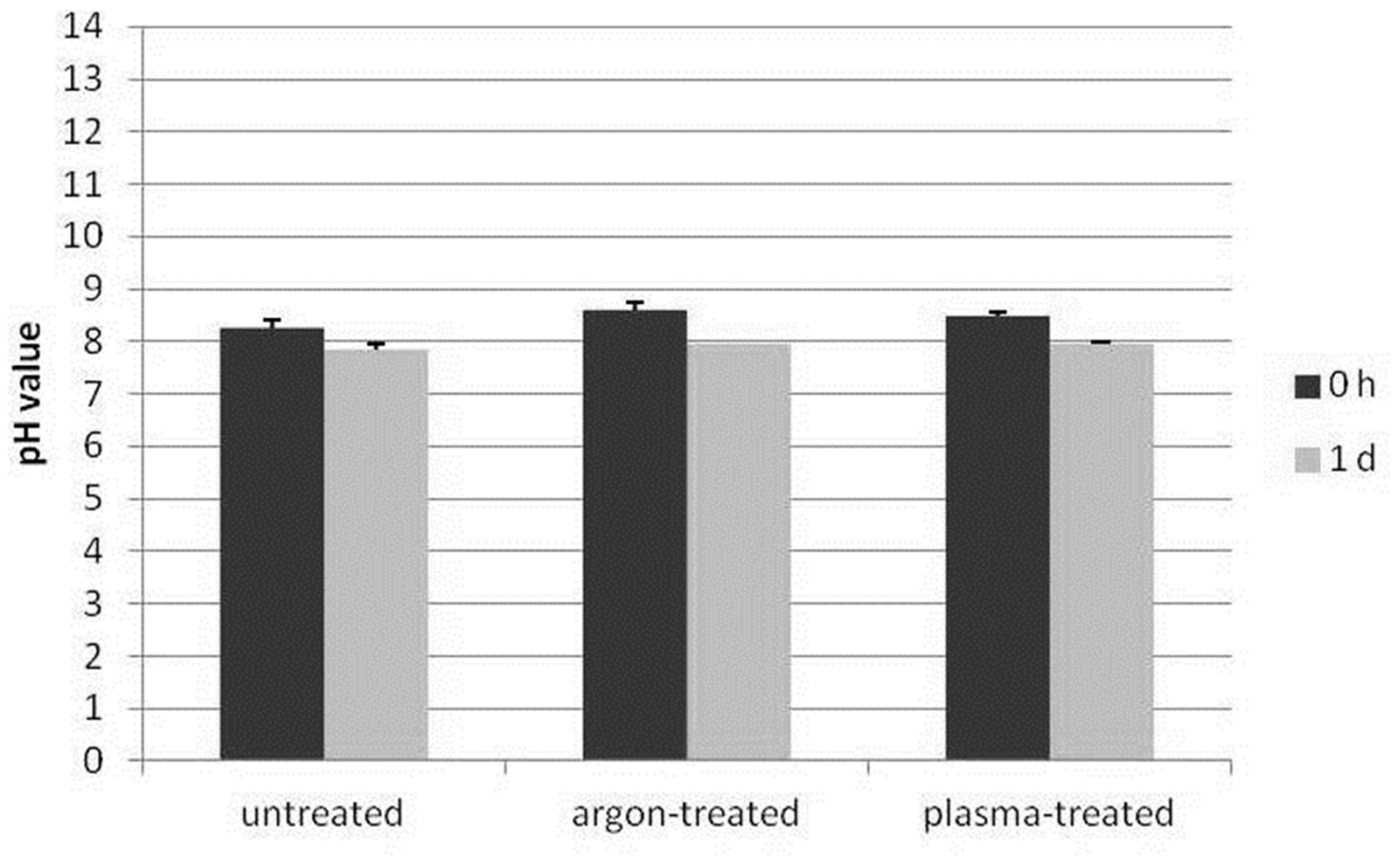
pH measurement of 60 s plasma-treated DMEM. No decisive pH change is to be seen 0 h and 1 d after plasma treatment. (n = 3).

**Figure 7:**
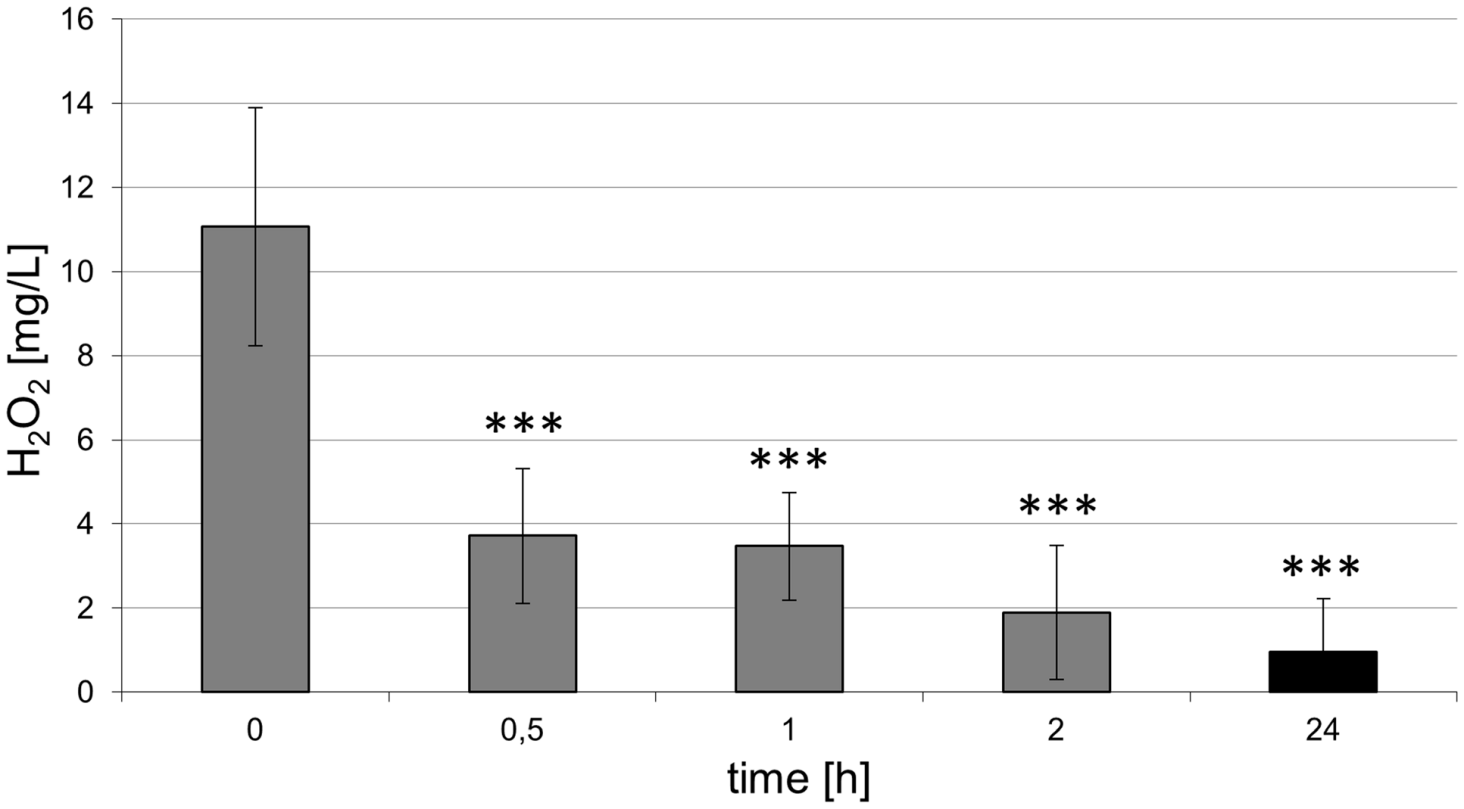
Concentration of hydrogen peroxide in 60 s plasma-treated DMEM. Note the continuous decrease of hydrogen peroxide over various time periods. (n = 7, Mann-Whitney *U*-test, ***p<0.001).

**Figure 8:**
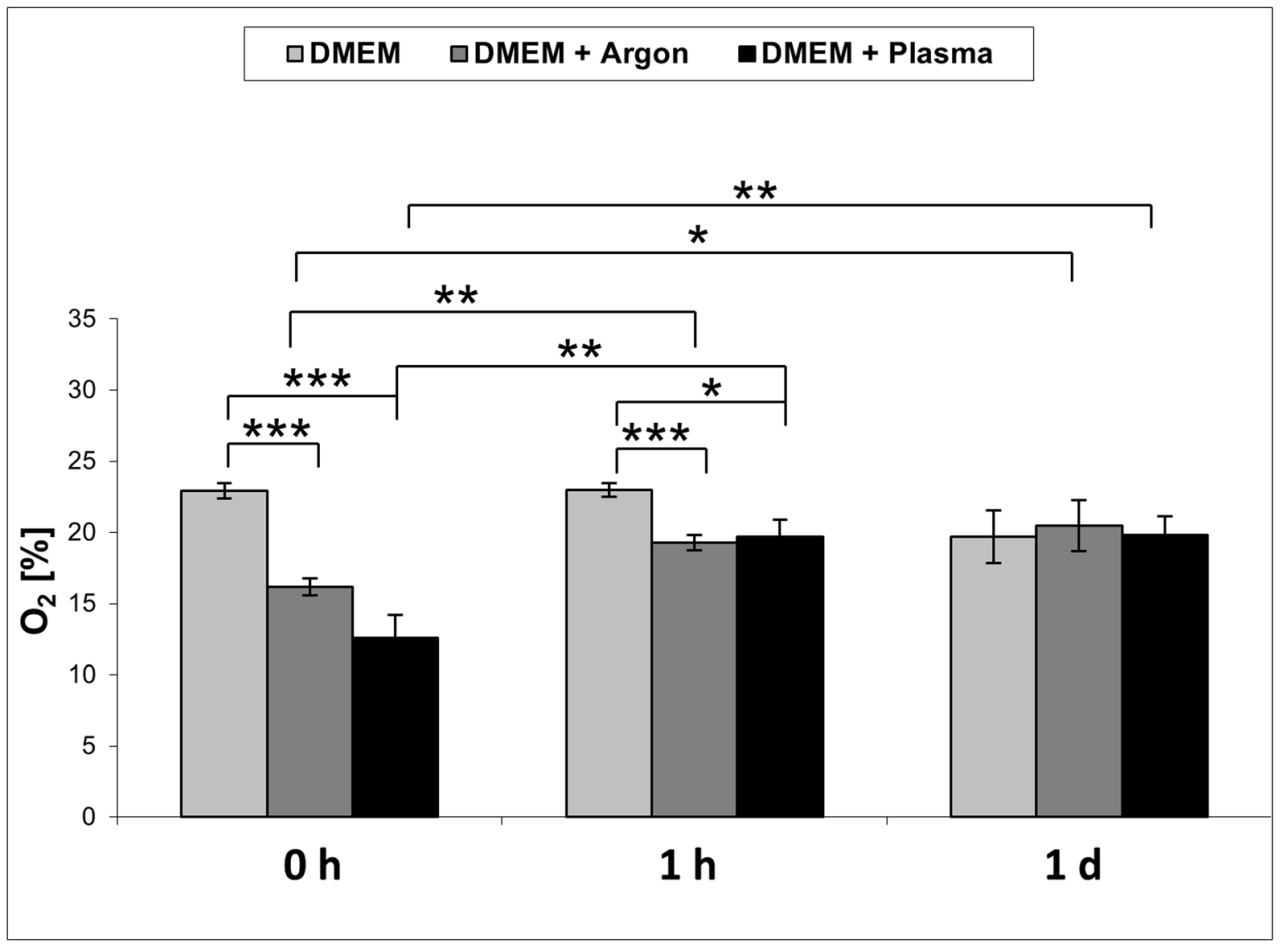
Oxygen content of 60 s plasma-treated DMEM. Note that in the plasma-treated complete DMEM the molecular oxygen concentration is decreased by half, which is equalised after only one day. (PreSens oxygen sensor, n = 3, student's *t*-test, *p<0.05, ** p<0.01, ***p<0.001).

Given the knowledge that reactive oxygen species, e. g. hydroxyl radicals, are generated in the plasma (see Figure 2) and these short-living species may influence the chemical composition of the cell culture medium DMEM, influential factors such as pH and hydrogen peroxide were determined. For the pH measurements, fresh, complete DMEM was plasma-treated for 60 s and the pH was determined directly after plasma treatment or 1 day after plasma treatment. The concentration of hydrogen peroxide was determined in 60 s plasma-treated DMEM for up to 1 day. As shown in **Figure 6**, the pH values of untreated *vs*. argon-treated (control) *vs.* plasma-treated DMEM directly (0 h) or 1 day after plasma treatment revealed no decisive pH changes. Although plasma treatment of DMEM immediately induced the formation of hydrogen peroxide as shown in **Figure 7**, the concentration decreases continuously over the time period up to 1 day. The oxygen content of the cell culture medium DMEM is seen in **Table 1** and **Figure 8**. It could be observed that plasma already decreased molecular oxygen concentration by half after plasma treatment of 60 s. Interestingly, after one day the oxygen content is equalised and therefore hypoxia may not contribute to the cellular effects observed after storing the medium for 7 days. However, treatment with pure argon gas also reduced the oxygen concentration. This is not surprising, because purging with argon is a usual method for oxygen elimination in solvents used for sensitive chemical reactions [45].

**Table 1:** Oxygen content of cell culture medium immediately after plasma treatment for 60 and 120 s. (PreSens oxygen sensor, mean ± SD, n = 3; student's *t*-test).

Treatment time	DMEM control (in %)	DMEM+Ar (in %)	DMEM+Plasma (in %)
**60 s**	22.92±0.54	16.16±0.59***	12.61±1.61***
**120 s**	21.74±3.17	14,71±3.12	10.90±2.18*

*p<0.05 *vs.* untreated,

***p<0.001 *vs.* untreated.

**Figure 9:**
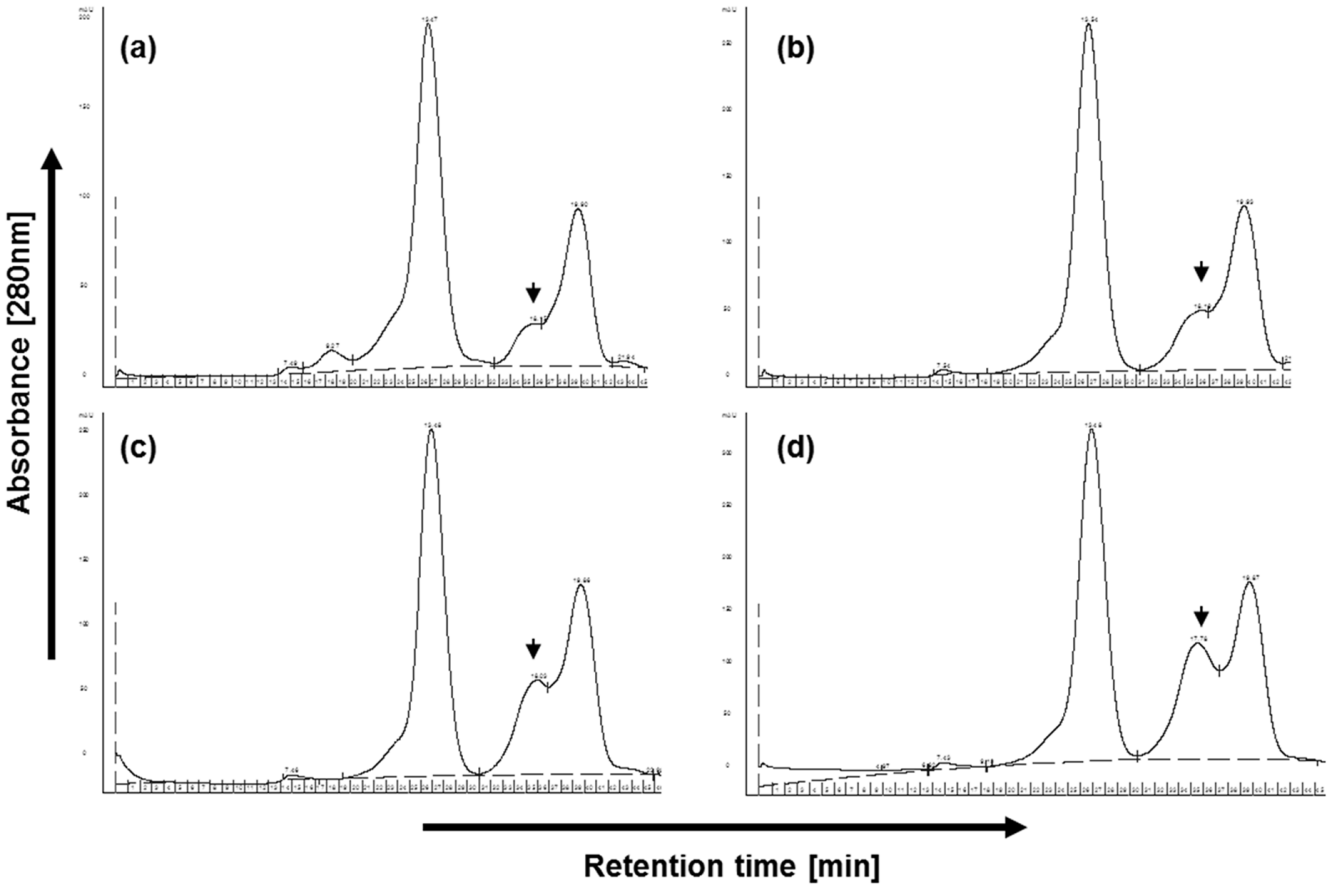
Representative gel filtration chromatogram resulting from: (a) application of untreated complete medium, (b) complete argon-gas treated medium, (c) complete medium with 60 s or (d) 120 s of plasma treatment. The arrowheads mark the one peak which alters considerably after plasma treatment. (n = 6 independent experiments).

Further investigations on the effect of plasma-treatment in medium revealed an increasing peak in gel filtration experiments (**Figure 9**). In these experiments we separated complete medium (DMEM+10% FCS) by gel filtration and detected an additional peak upon plasma treatment compared to argon gas treatment (as control). Interestingly, peak height increased dependent on the treatment time (see Figure 9c,d). This increase can be observed also in medium phenol red-free, antibiotics-free or serum-free (not shown). Whether this result is of importance for the effects of plasma treatment on cell viability awaits further analysis.

**Figure 10:**
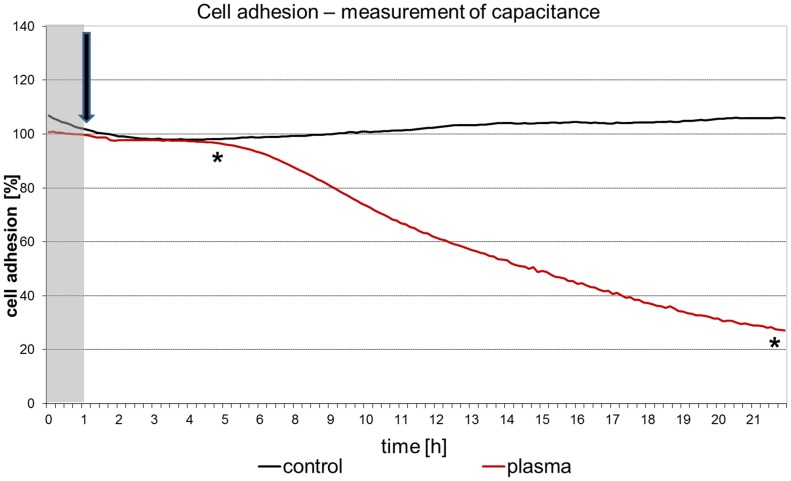
Online-monitoring of cell adhesion of living mHepR1 cells. Note that complete DMEM plasma-treated for 60 s, which was stored for 4 days at 37°C, induced the cells to detach from the sensor chip indicating the loss of adhesion capacity. The arrow indicates the time point of medium addition. (Bionas 1500 analysing system, n = 3, student's *t*-test * p<0.05 for all values from the time point of 4 hours).

**Figure 11:**
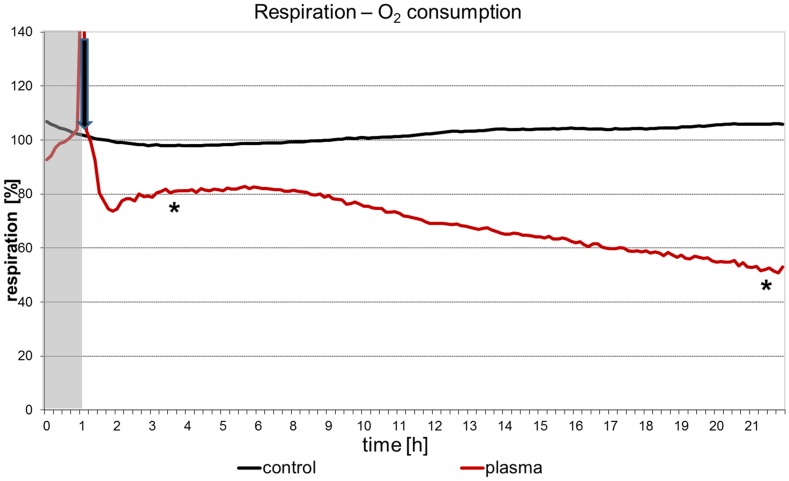
Online-monitoring of respiration of living mHepR1 epithelial cells. Note that the respiration is immediately disturbed after addition of complete DMEM plasma-treated for 60 s (arrow) which was stored for 4 days at 37°C. (Bionas 1500 analysing system, n = 3, student's *t*-test * p<0.05 for all values from the time point of 3.5 hours).

Online measurements of cell adhesion (**Figure 10**) and oxygen consumption (**Figure 11**) in vital, adherent mHep-R1 cells revealed that the effects can be seen at nearly all different time points gathered. The adhesion (investigated by impedance measurements) is not affected for roughly 5 hours after addition of the plasma-treated medium. After 6 h the cell adhesion decreases slightly, but steadily down to a value of 20%. In contrast, the respiration of the cells is blocked immediately after addition of the argon plasma-treated, 4 day stored medium. This decrease of about 20% remains constant for approx. 6 h. After this time point the respiration decreases further, down to 50% after 22 h. This slight decrease is in accordance with the decrease of cell adhesion.

## Discussion

An atmospheric pressure argon plasma jet [9] was used throughout the experiments. For the application of the plasma device, a characterisation was firstly performed. To eliminate plasma effects due to thermal damage to living matter or differential proteins in cell culture medium, the temperature was measured directly in the cell culture medium during a plasma treatment of up to 480 s. Temperatures over 40°C are to be regarded as critical in general [9]. The temperature remained almost constant up to a treatment time of 180 s. The cell culture medium did not exceed a temperature of 25°C and thus thermal damage could be excluded. Only with a treatment time of nearly 420 s a strong increase in temperature could be measured, caused by intense evaporation of the cell culture medium. Concerning evaporation - we could observe a slight liquid evaporation already after the 60 s treatment of 100 µl DMEM with argon plasma (n = 6, remaining volume mean = 75.6 µl). However, in the first experiment with evaporated DMEM (with argon gas, down to 75 µl) we could not find any effects on morphology/attachment (light microscopy) and vitality (via MTS-test) (C. Hoppe, personal communication).

Dependent on the process gas, the composition of the plasma radiation may vary. In *in vitro* experiments of Zhang *et al*.. [46], an emission spectrum of the plasma was recorded. Argon as the process gas and an admixture of 2.5% oxygen resulted in a high proportion of reactive oxygen and nitrogen species (ROS, RNS) and shares of ionised hydrogen (H_α_) in the spectral analysis. Using the pure process gas argon as in our experiments, a spectral analysis of the argon plasma revealed small amounts of hydroxyl radicals and molecular nitrogen. High levels of nitrogen species, as well as shares of ionised hydrogen could not be ascertained. Therefore, it can be assumed here that the reactive oxygen species can be assigned a key role as an active plasma component.

Adding oxygen, nitrogen or air to argon-operated atmospheric pressure plasmas leads to enhanced production of ROS and RNS, respectively [46], [47]. In the present case, the plasma jet was operated with pure argon; nevertheless, optical emission spectroscopy revealed the presence of hydroxyl radicals and atomic oxygen. It has been shown on a kINPen plasma source that ambient oxygen and nitrogen surrounding the effluent can be a source of ROS/RNS in a cellular plasma treatment [48]. We can assume that these species are also present in our case. The presence of OH emission bands reveals that water dissociation occurs within the plasma zone. This humidity can originate from feed gas impurities and can also contribute significantly to biological effects [39].

A biological liquid-mediated effect of plasma treatment was observed for the first time by our group on tight junctions, jointly responsible for cell-cell connections [34]. Large openings were observed which led to the complete degradation of the cell connections. Surprisingly, in the present work cell culture medium which was plasma treated and then stored for more than seven days was still effective in inducing these cell morphological changes. A retraction of the ZO-1 protein from the cell membrane occurred which was accompanied by an increased degradation of ZO-1. Going into this work in detail, the immunostaining of the zonula occludens (ZO-1) tight junction protein in untreated mHepR1 cells revealed that ZO-1 is localised at the cell margin, expressing continuous ZO-1 bands which connect the adjacent cells. The ZO-1 protein is located in the submembranous domain of the tight junctions and is associated *via* the transmembrane proteins occludin and claudin with the actin cytoskeleton [49]. Due to this, the cells are strengthened in their stability and relate to their neighboring cells in a closely connected way. Other authors described that pharmacological substances led to changes in the protein composition and expression and local distribution of tight junctions, as observed in epithelial Madine-Darby canine kidney cells by Bojarski *et al.* [50]. These cells were treated with the protein kinase inhibitor staurosporine to artificially initiate apoptosis. In addition, the continuous ZO-1 bands disappeared and partially joined to form an irregular distribution of the ZO-1 in the cytoplasm [50]. Similar results have been published in connection with calcium deficiency and induction of hypoxia [51], [52]. You *et al.* [53] observed a disruption of the pulmonal epithelial barrier in neonatal animals induced by hyperoxia, which is, at least in part, due to massive deterioration in the expression and localisation of key tight junction proteins. Tight junction openings were also observed in human lens epithelial cells *in vitro* after incubation with a T-calcium channel inhibitor (10–20 µM Mibefradil) [54]. Due to this inhibitor an increase in apoptosis was observed, but not in proliferation. However, the carcinogenic potential of the cells after tight junction openings remains to be investigated.

Although there is increasing evidence that plasma treatment promotes the healing process of tissue and accelerates wound healing [18]–[21], [55], however the data reported here put forward the idea that argon plasma-treated liquids alter epithelial cell monolayer and impair cell adhesion. These are opposite evidences and should be discussed. This point can be interpreted in two ways. (i) Sensitivity of *in vitro vs. in vivo*: concerning wound healing the skin is a complex architectural multilayer and not comparable with an in vitro epithelial monolayer presented here. We know from in vitro *vs.* in vivo approaches using copper as anti-microbial agents on implant surfaces that the cell monolayer in vitro is much more sensitive to agents because there are no cells in the “second row” to protect or replace the apical cell row. Thus, Hoene *et al.* [56] reported that copper coated titanium caused solely a moderately increased local inflammatory response in the rat musculature. In contrast, other studies in vitro revealed strong cytotoxicity of titanium-copper in an osteoblast monolayer [57]. (ii) Penetration depth: providing that opening of cell-cell contacts by plasma treatment also function in vivo – and hints for that are found by Fluhr *et al.* [58] (due to tissue-tolerable electrical plasma increase in transepidermal water loss in vivo indicating an impaired skin barrier function) – the openings of tight junctions could have a positive effect for the penetration of conventional therapeutics (antibiotics, disinfectants) on skin. Often, the application of conventional liquid antiseptics is not sufficient and sustainable as the borders and the surrounding of chronic wounds frequently consist of sclerotic skin, impeding an effectual penetration of these products [59].

The formations of cell surface structures, e. g. microvilli are essential for characteristic functions of specialised cells in tissues. Microvilli primarily serve to increase the cell surface for an optimal metabolite exchange and, moreover, microvilli are responsible for the coordination between glycolysis and oxidative phosphorylation, regulation of cellular functions by external signals, as well as for Ca^2+^ signaling [60]. Our morphological investigations of the mHepR1 cell surface using scanning electron microscopy revealed shortened microvilli with a lower density or rather a loss of microvilli after plasma treatment. A global loss of microvilli on alveolar cells was also detected in the work of Niset *et al.* [61] when they performed studies on bacterial infections of the respiratory tract promoted by pneumonia or lung injury. Besides bacterial causes, also various toxins induce cell membrane damage accompanied by the loss of microvilli. As early as 1976 Pfister and Burstein [62] studied the effect of ophthalmic drugs, e. g. neopolycin, benzalkonium chloride, on corneal epithelial cells. They observed the loss of surface epithelial microvilli as well as rupture of the tight junctions. These observations are similar to our results presented here after incubating cells with plasma-treated and long-time stored medium, though the effect of plasma treatment on cell surfaces has hitherto not been examined.

Therefore, it was clear that the plasma effect is not only mediated by liquids but is also long-lasting in its effectivity. Considering these observations, it was assumed that plasma-induced changes in medium components are responsible. For this reason, a first analysis of the cell culture medium DMEM after plasma exposure was carried out. In our experiments the pH value remained constant, as expected. The buffering capacity of sodium hydrogen carbonate in the cell culture medium was sufficient to maintain the pH even after a plasma treatment.

Plasma-liquid interactions were studied in the work of Oehmigen *et al*.. [29] and von Woedtke *et al*.. [30] relating to the effective disinfection of liquids. In their studies the inactivation of bacteria is strongly dependent on the acidification of aqueous liquids (pH decrease). Beyond the finding that the inactivation of bacteria is dependent on acidic liquids, the bactericidal efficacy is increased by plasma-generated chemical species, e. g. nitrate and hydrogen peroxide. In particular, the redox amphoteric hydrogen peroxide was found in liquids after plasma treatment in various studies [7], [63], [64]. Hydrogen peroxide also occurs in the cell metabolism of oxygen as a by-product and can be degraded in the cell by various repair and protection mechanisms. However, hydrogen peroxide reacts with bivalent iron in the Fenton-reaction to generate hydroxyl radicals and trivalent iron [65]. This formation of hydroxyl radicals could be enhanced by an additional amount of hydrogen peroxide induced by plasma treatment. So hydrogen peroxide was detected in high concentrations, in particular directly after plasma treatment. The disturbances in the redox state cause oxidation of lipids, proteins and nucleic acids in their surroundings, which may lead to lethal cell damage. These possible plasma effects in interaction with living matter have already been described [66]. In our work, the measurements of the concentrations of hydrogen peroxide showed that hydrogen peroxide strongly decreased even after two hours and it was no longer detectable one day after plasma treatment for 60 s. Hydrogen peroxide tends to rapidly decompose into water and molecular oxygen [67]. This decomposition reaction can be accelerated, especially by ferrous catalysts, where more molecular oxygen is released from liquids. A catalysed decomposition of hydrogen peroxide can be carried out *via* iron nitrate, which is found in the cell culture medium DMEM.

In a recent paper, Kalghatgi *et al*.. [35] demonstrated an induction of DNA damage in mammalian breast epithelial cells by a plasma-treated cell culture medium (DMEM). This effect was not reduced if the medium was stored up to one hour prior to addition to the cells. The authors hypothesised that this retained biological effect of a plasma-treated cell culture medium is caused by the formation of stable organic peroxides from medium compounds like amino acids [35]. Formation of stable peroxides from amino acids and proteins by ROS like the OH radical is a known process [68]. In recent years, an active discussion has started about the truly complex ROS chemistry in plasma-treated liquids, including reactive species like the OH radical, hyperoxide anion, and also the relatively stable hydrogen peroxide and its biological effects [28], [29], [47], [69], [70]–[72]. Both the persisting biological effect in plasma-treated cell culture medium up to seven days and the strongly decreasing hydrogen peroxide concentration in the medium during the first day after plasma treatment as presented in this paper could support the hypothesis of the formation of stable organic peroxides in a cell culture medium by plasma-induced ROS chemistry. In future studies on long-time stored liquids we have to shed light on the question which stable liquid components influence cell physiology and morphology.

## Conclusions

The efficacy of plasma-treated complete cell culture medium DMEM stored for one week and its impact on the cell physiology of epithelial mHepR1 cells was ascertained. We discovered that the liquid-mediated effect of atmospheric pressure argon plasma on epithelial cells persists through up to one week of storage. Although our first chemical analyses revealed that temperature and pH (both were constant), hydrogen peroxide production and oxygen content (both decrease within one day) can be excluded as initiators of cellular changes after one week, cells growing in a monolayer were attacked. We observed a loss of cell-cell contacts and cell adhesion accompanied by a reduced cell respiration capacity. The microvilli on the cell membrane lost their intensity of expression. Both the persisting biological effect in a plasma-treated cell culture medium and the strongly decreasing hydrogen peroxide concentration in the medium during the first day after plasma treatment could support the hypothesis of the formation of stable organic peroxides in a cell culture medium by plasma-induced ROS chemistry.

The here observed persisting biological effect in plasma-treated liquids could open new medical applications. The vision could be the establishment of local plasma centers to prepare relatively stable plasma-treated liquids for dermatological (chronical wounds, tumors), dental (periimplantitis) or orthopaedic (joint rinsing) applications to support or replace the conventional therapy.

## References

[1] Fridman G, Friedman G, Gutsol A, Shekhter AB, Vasilets VN, et al (2008) Applied plasma medicine. Plasma Process Polym 5: 503–533.

[2] Vasilets VN, Gutsol A, Shekhter AB, Fridman A (2009) Plasma medicine. High Energy Chem 43: 3.

[3] Lloyd G, Friedman G, Jafri S, Schultz G, Fridman A, et al (2009) Medical uses and developments in wound care. Plasma Process Polym 7: 194–211.

[4] Moreau M, Orange N, Feuilloley MGJ (2008) Non-thermal plasma technologies: new tools for bio-decontamination. Biotechnol Adv 26: 610–617. 18775485 10.1016/j.biotechadv.2008.08.001

[5] Daeschlein G, von Woedtke Th, Kindel E, Brandenburg R, Weltmann K-D, et al (2010) Antibacterial activity of an atmospheric pressure plasma jet against relevant wound pathogens in vitro on a simulated wound environment. Plasma Process Polym 7: 224–230.

[6] Daeschlein G, Scholz S, Ahmed R, von Woedtke T, Haase H, et al (2012) Skin decontamination by low-temperature atmospheric pressure plasma jet and dielectric barrier discharge plasma. J Hosp Infect 81 (3) 177–183. 22682918 10.1016/j.jhin.2012.02.012

[7] Nosenko T, Shimizu T, Morfill GE (2009) Designing plasmas for chronic wounds disinfections. New J Phys 11: 1–19.

[8] Nastuta AV, Topala I, Grigoras C, Pohoata V, Popa G (2011) Stimulation of wound healing by helium atmospheric pressure plasma treatment. J Phys D: Appl Phys 44: 10.

[9] Weltmann K-D, Kindel E, Brandenburg R, Meyer C, Bussiahn R, et al (2009) Atmospheric pressure plasma jet for medical therapy: plasma parameters and risk estimation. Contr Plasma Physics 49: 631–640.

[10] Plewa JM, Yousfi M, Frongia C, Eichwald O, Ducommun B, et al (2014) Low-temperature plasma-induced antiproliferative effects on multi-cellular tumor spheroids. New J Phys 16: 043027.

[11] Ishaq M, Evans M, Ostrikov K (2014) Effect of atmospheric gas plasmas on cancer cell signaling. Int J Cancer 134: 1517–1528. 23754175 10.1002/ijc.28323

[12] Graves DB (2012) The emerging role of reactive oxygen and nitrogen species in redox biology and some implications for plasma applications to medicine and biology. J Phys D: Appl Phys 45: 263001.

[13] Brandenburg R, Lange H, v Woedtke Th, Stieber M, Kindel E, et al (2009) Antimicrobial effects of UV and VUV radiation of nonthermal plasma jets. IEEE Trans Plasma Sci 37: 877–883.

[14] Kim SJ, Chung TH, Bae SH, Leem SH (2009) Bacterial inactivation using atmospheric pressure single pin electrode microplasma jet with a ground ring. IEEE Trans Plasma Sci 94: 141502–4.

[15] Fröhling A, Baier M, Ehlbeck J, Knorr D, Schlüter O (2012) Atmospheric pressure plasma treatment of Listeria innocua and Escherichia coli at polysaccharide surfaces: Inactivation kinetics and flow cytometric characterization. Innov Food Sci Emerg Technol 13: 142–150.

[16] Ermolaeva S, Varfolomeev AF, Chernukha MY, Yurov DS, Vasiliev MM, et al (2011) Bactericidal effects of non-thermal argon plasma in vitro, in biofilms and in the animal model of infected wounds. J Med Microbiol 60: 75–83. 20829396 10.1099/jmm.0.020263-0

[17] Barekzi N, Laroussi M (2012) Dose-dependent killing of leukemia cells by low-temperature plasma. J Phys D: Appl Phys 45: 422002.

[18] Heinlin J, Morfill G, Landthaler M, Stolz W, Isbary G, et al (2010) Plasma medicine: possible applications in dermatology. J Dtsch Dermatol Ges 8: 968–976. 20718902 10.1111/j.1610-0387.2010.07495.x

[19] Heinlin J, Isbary G, Stolz W, Morfill G, Landthaler M, et al (2011) Plasma applications in medicine with a special focus on dermatology. J Europ Academy Dermat Venereol 25: 1–11. 10.1111/j.1468-3083.2010.03702.x 20497290

[20] Stoffels E (2008) Atmospheric plasma: a universal tool for physicians? Low Temperature Plasmas: Fundamentals, Technologies and Techniques 2, ed. Hippler R, Kersten H, Schmidt M, Schoenbach KH, 675, Wiley-VCH, Berlin. pp. 1–30.

[21] Daeschlein G, Scholz S, Ahmed R, Majumdar A, von Woedtke T, et al (2012a) Cold plasma is well-tolerated and does not disturb skin barrier or reduce skin moisture. J Dtsch Dermatol Ges 10 (7) 509–515. 22405534 10.1111/j.1610-0387.2012.07857.x

[22] Diegelmann RF, Evans MC (2004) Wound healing: an overview of acute, fibrotic and delayed healing. Front Biosci 1;9: 283–289. 10.2741/1184 14766366

[23] Velnar T, Bailey T, Smrkolj V (2009) The wound healing process: an overview of the cellular and molecular mechanisms. J Int Med Res 37 (5) 1528–1542. 19930861 10.1177/147323000903700531

[24] Knittel T, Dinter C, Kobold D, Neubauer K, Mehde M, et al (1999) Expression and regulation of cell adhesion molecules by hepatic stellate cells (HSC) of rat liver: involvement of HSC in recruitment of inflammatory cells during hepatic tissue repair. Am J Pathol 154 (1) 153–167. 9916930 10.1016/s0002-9440(10)65262-5 PMC1853435

[25] Clark EA, Brugge JS (1995) Integrins and signal transduction pathways: the road taken. Science 268: 233–239. 7716514 10.1126/science.7716514

[26] Ekblom P, Timpl R (1996) Cell-to-cell contact and extracellular matrix. A multifacted approach emerging. Curr Opin Cell Biol 8: 599–601. 8939665 10.1016/s0955-0674(96)80099-8

[27] Haertel B, Strassenburg S, Oehmigen K, Wende K, von Woedtke Th, et al (2013) Differential influence of components resulting from atmospheric-pressure plasma on integrin expression of human HaCaT keratinocytes. BioMed Res Intern 2013: 761451. 10.1155/2013/761451 PMC3712198 23936843

[28] von Woedtke Th, Reuter S, Masur K, Weltmann K-D (2013) Plasmas for medicine. Physics Reports 530: 291–320.

[29] Oehmigen K, Winter J, Hähnel M, Wilke C, Brandenburg R, et al (2011) Estimation of possible mechanisms of escherichia coli inactivation by plasma treated sodium chloride solution. Plasma Process Polym 8: 904–913.

[30] von Woedtke Th, Oehmigen K, Brandenburg R, Hoder T, Wilke C, et al.. (2012) Plasma-liquid-interactions: chemistry and antimicrobial effects. In: ZMachala, KHensel,YAkishev, editors: Plasma for bio-decontamination, medicine and food security, NATO Science for Peace and Security Series–A: Chemistry and Biology, Springer, Dordrecht. pp. 67–78.

[31] Traylor MJ, Pavlovich MJ, Karim S, Hait P, Sakiyama Y, et al (2011) Long-term antibacterial efficacy of air plasma-activated water. J Phys D: Appl Phys 44: 472001.

[32] Naïtali M, Kamgang-Youbi G, Herry J-M, Bellon-Fontaine M-N, Brisset J-L (2010) Combined effects of long-living chemicals during microbial inactivation using atmospheric plasma-treated water. Appl Environ Microbiol 76: 7662–7664. 20889799 10.1128/AEM.01615-10 PMC2976197

[33] Julak J, Scholtz V, Kotucova S, Janouskova O (2012) The persistent microbicidal effect in water exposed to the corona discharge. Physica Medica 28: 230–239. 21925912 10.1016/j.ejmp.2011.08.001

[34] Hoentsch M, von Woedtke T, Weltmann K-D, Nebe B (2012) Time-dependent effects of low-temperature atmospheric-pressure argon plasma on epithelial cell attachment, viability and tight junction formation in vitro. J Physics D: Appl Physics 45: 025206.

[35] Kalghatgi S, Kelly CM, Cerchar E, Torabi B, Alekseev O, et al (2011) Effects of non-thermal plasma on mammalian cells. PLoS One 6: 16270. 10.1371/journal.pone.0016270 PMC3025030 21283714

[36] Haertel B, Haehnel M, Blackert S, Wende K, von Woedtke T, et al (2012) Surface molecules on HaCaT keratinocytes after interaction with non-thermal atmospheric pressure plasma. Cell Biol Int 36: 1217–1222. 22973947 10.1042/CBI20120139

[37] Bundscherer L, Wende K, Ottmüller K, Barton A, Schmidt A, et al (2013) Impact of non-thermal plasma treatment on MAPK signaling pathways of human immune cell lines. Immunobiol 218: 1248–1255. 10.1016/j.imbio.2013.04.015 23735483

[38] Schmidt A, Wende K, Bekeschus S, Bundscherer L, Barton A, et al (2013) Non-thermal plasma treatment is associated with changes in transcriptome of human epithelial skin cells. Free Radic Res 47: 577–592. 23668811 10.3109/10715762.2013.804623

[39] Winter J, Wende K, Masur K, Iseni S, Dünnbier M, et al (2013) Feed gas humidity: a vital parameter affecting a cold atmospheric-pressure plasma jet and plasma-treated human skin cells. J Phys D: Appl Phys 46: 295401.

[40] Nebe B, Rychly J, Knopp A, Bohn W (1995) Mechanical induction of beta1-integrin mediated calcium signaling in a hepatocyte cell line. Exp Cell Res 218: 479–484. 7540984 10.1006/excr.1995.1181

[41] Rebl H, Finke B, Schroeder K, Nebe JB (2010) Time-dependent metabolic activity and adhesion of human osteoblast-like cells on sensor chips with a plasma polymer nanolayer. Int J Artif Organs 33: 738–748. 21058265

[42] Ehret R, Baumann W, Brischwein M, Schwinde A, Stegbauer K, et al (1997) Monitoring of cellular behavior impedance measurements on interdigitated electrode structures. Biosens Bioelectron 12,1: 29–41. 8976050 10.1016/0956-5663(96)89087-7

[43] Bradford MM (1976) A rapid and sensitive method for the quantitation of microgram quantities of protein utilizing the principle of protein-dye binding. Anal Biochem 72: 248–254. 942051 10.1016/0003-2697(76)90527-3

[44] Wende K, Straßenburg S, Haertel B, Harms M, Holtz S, et al (2014) Atmospheric pressure plasma jet treatment evokes transient oxidative stress in HaCaT keratinocytes and influences cell physiology. Cell Biol Int 38 (4) 412–425. 24155089 10.1002/cbin.10200

[45] Battino R, Clever HL (1966) The solubility of gases in liquids. Chem Rev 66 (4) 395–463.

[46] Zhang X, Li M, Zhou R, Feng K, Yang S (2008) Ablation of liver cancer cells in vitro by a plasma needle. Appl Phys Lett 93: 021502–1.

[47] Pipa AV, Reuter S, Foest R, Weltmann K-D (2012) Controlling the NO production of an atmospheric pressure plasma jet. J Phys D: Appl Phys 45: 085201.

[48] Reuter S, Tresp H, Wende K, Hammer MU, Winter J, et al (2012) From RONS to ROS: tailoring plasma jet treatment of skin cells. IEEE Trans Plasma Sci 40: 2986–2993.

[49] Orbán E, Szabό E, Lotz G, Kupcsulik P, Páska C, et al (2008) Different expression of occludin and ZO-1 in primary and metastatic liver tumors. Pathol Oncol Res 14: 299–306. 18386163 10.1007/s12253-008-9031-2

[50] Bojarski C, Weiske J, Schöneberg T, Schröder W, Mankertz J, et al (2003) The specific fates of tight junction proteins in apoptotic cells. J Cell Sci 117: 2097–2107. 10.1242/jcs.01071 15054114

[51] Siliciano JD, Goodenough DA (1988) Localization of the tight junction protein, ZO-1, is modulated by extracellular calcium and cell-cell contact in Madin-Darby canine kidney epithelial cells. J Cell Biol 107: 2389–2389. 3058722 10.1083/jcb.107.6.2389 PMC2115673

[52] Kimura K, Teranishi S, Kawamoto K, Nishida T (2009) Protection of human epithelial cells from hypoxia-induced disruption of barrier function by hepatocyte growth factor. Exp Eye Res 9: 337–343. 10.1016/j.exer.2009.11.012 19944686

[53] You K, Xu X, Fu J, Xu S, Yue X, et al (2012) Hyperoxia disrupts pulmonary epithelial barrier in newborn rats via the deterioration of occludin and ZO-1. Respir Res 13: 36. 22559818 10.1186/1465-9921-13-36 PMC3424121

[54] Weidmann A, Meissner A, Beck R, Nebe B (2008) Sensitivity of lens epithelial cells to mibefradil depends on the state of confluence. Eur J Cell Biol 87S1 (Suppl. 58) 75.

[55] Emmert S, Brehmer F, Hänßle H, Helmke A, Mertens N, et al (2013) Atmospheric pressure plasma in dermatology: Ulcus treatment and much more. Clinical PlasmaMedicine 1: 24–29.

[56] Hoene A, Prinz C, Walschus U, Lucke S, Patrzyk M, et al (2013) In vivo evaluation of copper release and acute local tissue reactions after implantation of copper-coated titanium implants in rats. Biomed Mater 2013 Jun;8 (3) 035009. 10.1088/1748-6041/8/3/035009 23598370

[57] Stranak V, Rebl H, Zietz C, Arndt K, Bogdanowicz R, et al (2011) Deposition of Titanium-Copper Coatings with Antimicrobial Effect by Advanced Magnetron Sputtering Methods. Mater Sci Engin C 31 (2011) 1512–1519.

[58] Fluhr JW, Sassning S, Lademann O, Darvin ME, Schanzer S, et al (2012) In vivo skin treatment with tissue-tolerable plasma influences skin physiology and antioxidant profile in human stratum corneum. Exp Dermatol 21 (2) 130–134. 22142271 10.1111/j.1600-0625.2011.01411.x

[59] Lademann O, Kramer A, Richter H, Patzelt A, Meinke MC, et al (2011) Skin disinfection by plasma-tissue interaction: comparison of the effectivity of tissue-tolerable plasma and a standard antiseptic. Skin Pharmacol Physiol 24 (5) 284–288. 21709431 10.1159/000329913

[60] Lange K (2002) Role of microvillar cell surfaces in the regulation of glucose uptake and organization of energy metabolism. Am J Physiol Cell Physiol 282 (1) C1–26. 11742794 10.1152/ajpcell.2002.282.1.C1

[61] Nizet V, Gibson RL, Chi EY, Framson PE, Hulse M, et al (1996) Group B streptococcal beta-hemolysin expression is associated with injury of lung epithelial cells. Infect Immun 6: 3818–3826. 10.1128/iai.64.9.3818-3826.1996 PMC174298 8751934

[62] Pfister RR, Burstein N (1976) The effects of ophthalmic drugs, vehicles, and preservatives on corneal epithelium: a scanning electron microscope study. Investig Ophthalmol Vis Sci 15: 246–259. 1262158

[63] Oehmigen K, Hähnel M, Brandenburg R, Wilke Ch, Weltmann K-D, et al (2010) The role of acidification for antimicrobial activity of atmospheric pressure plasma in liquids. Plasma Process Polym 7: 250–257.

[64] Shainsky N, Dobrynin D, Ercan U, Joshi S, Ji H, et al. (2011) Non-equilibrium plasma treatment of liquids, formation of plasma acid. www.ispc-conference.org/ispcproc/ispc20/701.pdf

[65] Wenner J (2006) The role of oxygen radicals in the regulation of hypoxia-inducible factors. PhD Thesis, Johann Wolfgang Goethe University Frankfurt am Main.

[66] Dobrynin D, Fridman G, Friedman G, Fridman A (2009) Physical and biological mechanism of direct plasma interaction with living tissue. New J Phys 11: 1–6.

[67] Riedel E, Janiak C (1999) Allgemeine und Anorganische Chemie, 7^th^ edition, Walter de Gruyter, Berlin New York, ISBN 978-3-11-018903-2.

[68] Gebicki S, Gebicki J M (1993) Formation of peroxides in amino acids and proteins exposed to oxygen free radicals. Biochem J 289: 743–749. 8435071 10.1042/bj2890743 PMC1132237

[69] Lukes P, Locke BR, Brisset J-L (2012) Aqueous-phase chemistry of electrical discharge plasma in water and in gas-liquid environments. Plasma chemistry and catalysis in gases and liquids. ed. Parvulescu VI, Magureanu M, Lukes P WILEY-VCH, Weinheim pp. 243–308.

[70] van Gils CAJ, Hofmann S, Boekema BKHL, Brandenburg R, Bruggeman PJ (2013) Mechanisms of bacterial inactivation in the liquid phase induced by a remote RF cold atmospheric pressure plasma jet. J Phys D: Appl Phys 46: 175203.

[71] Machala Z, Tarabova B, Hensel K, Spetlikova E, Sikurova L, et al (2013) Formation of ROS and RNS in water electro-sprayed through transient spark discharge in air and their bactericidal effect. Plasma Process Polym 10: 649–659.

[72] Stoffels E, Sakiyama Y, Graves DB (2008) Cold atmospheric plasma: charged species and their interactions with cells and tissues. IEEE Trans Plasma Sci 36: 4.

